# Medical Miscellanies

**Published:** 1816-07

**Authors:** 


### PART IV.

### MEDICAL MISCELLANIES.

### FOREIGN and DOMESTIC.

*An Account of the following singular Phænomenon, recently observed on Mount Etna, has been communicated by Signor Mario Gemmellaro, of Catania, to his brother, a Student of Medicine, at Paris.*—" Two Englishmen went yesterday to visit Mount Etna; and witnessed a phænomenon completely new to ourselves and our natural philosophers.

" The atmosphere near the summit of the mountain was loaded with clouds, which threatened a hail-storm; and as our little habitation* was yet covered with snow, the travellers hastily descended the mountain. On arriving near Mount Frumento, they observed flashes of lightning beneath their feet, (a circumstance not uncommon in these regions) and the column of air which now surrounded them, became sonorous in the most curious and striking manner.

* Signor Gemmellaro has erected a small building on Mount Etna, at his own expence, for the accommodation of travellers.

"Our three travellers walked upon the snow; their clothes were damp, and their progressive motion produced a constant sound; it was uniform, monotonous, and continued; resembled a hissing or piercing noise, and was audible at the distance of a hundred paces. Their heads became affected with stupor, as tho' the forehead and temples were encircled by a bandage; and the hair stood erect like hog's bristles.

"The Guide imagined that he felt a swarm of horse flies under his cap, and lifted up his hand to drive them away; but on raising it vertically, a stronger and more acute sound was excited. One of the Englishmen, observing this phænomenon, elevated his arm yet higher: a sound still more acute succeeded; and again, by moving his fingers in the air, he produced musical sounds. The Guide also obtained a similar result, sometimes with his right han', sometimes with his left; but, on employing both, no sound was elicited. The other Englishman could produce these sounds with his right hand, but not at all with his left.

"Upon proceeding some paces farther, the travellers found themselves no longer in the column of sonorous air; and nothing particular was observed. On re-entering it, however, the same musical sounds were produced. After some minutes, the sonorous column dispersed, and every trace of the phænomenon was lost.

"The English travellers promised me to consult the natural philosophers of their country upon this singular occurrence, and afterwards to publish the relation of it. I request that you will also consult the scientific men of your acquaintance, and send me their explanation. I shall give an account of this phænomenon in my intended work on the Phænomena of Etna." *Gazette de Santé, April* 1816.

---

*Electrical Phænomenon observed in Spain.*—Some of the Spanish monks are constantly in the habit of wearing flannel next the skin. In winter, they have stockings of the same materials; so that they are nearly isolated; and communicate with the atmosphere only by the head. This circumstance sometimes serves to acquire for them great consideration. The continual friction of the body with the flannel, produces an extrication of electric fluid; to which the head alone serves as conductor in its course to the general reservoir: hence when, in cold and dry weather, the monks, before day-break, quit their cells to repair to church, their heads appear to be surrounded by a luminous circle, which inspires, in ignorant spectators, the idea of something supernatural. The custom of representing the inhabitants of the celestial abodes with their heads surrounded by a circle of light, is, perhaps, indebted for its origin to the observation of a similar fact; necessarily inexplicable in an age when natural philosophy was yet an infant science. *Ardouin, Journal de Medécine, par Leroux.*

*Poisoning by Verdigris.*—Twelve soldiers having macerated a piece of meat with vinegar and spices in a copper saucepan badly tinned, made it the principal part of their meal on the succeeding day. On leaving the table, they all experienced vomiting, violent colic, cold sweats, &c. Seven of these men died; the other five, having probably eaten less heartily, escaped with colics and gripes. But one of these, who had lost the membrum virile from syphilis, was attacked with most intolerable Thirst. Irritated to fury whenever he wanted water, or solid food was presented to him, he died in extreme Marasmus on the fiftieth day from the ingestion of the poison. The urine of this unfortunate man was destitute of colour or taste; and he drank it without repugnance, whenever water or other liquids were refused him. On dissection of the body of this man, the palate, pharynx, and superior part of the œsophagus, were found completely inflamed. The gall bladder was filled with bile of a very bright colour; and destitute of decided bitterness." *Marchal, Journal de Medecine, &c. par Leroux.*

*Injurious Effects of recent Gratiola (Hedge Hyssop) employed in Glysters.*—A late Number of a French Journal contains the narrative of four cases, in which the employment of this plant, as an injection, was followed by decided symptoms of Furor Uterinus. All the subjects seem to have been women of a sanguine temperament. We shall transcribe one of the cases, which appears to us most interesting. "A lady, aged nineteen, attacked by scrofulous symptoms, took with advantage, for some months, a composition of gratiola and digitalis; after which, the employment of injections of strong decoction of gratiola was recommended. The first, which was thrown up, caused the evacuation of a great quantity of *thick and even compact slime.* The second produced intolerable itching of the vulva. After the third, the evacuations resembled scrapings of the intestines; and the itching was aggravated. Lastly, the fourth excited severe griping and copious discharges, followed by palpitations, heat in the abdomen, and all the symptoms of genuine Nymphomania. This affection was cured in three weeks by proper treatment; but the patient, haunted by recollection of the excesses to which she had been driven, terminated her existence by suicide." *Bibliothèque Medicale, January* 1816.

*New Bandage for transverse Wounds of the Throat.*—M. Cavalier, a French army surgeon, sometime since, proposed a bandage for wounds of the throat; which we think well adapted to the purpose; and of which we shall endeavour to give a description. Its successful application is illustrated by the case of a soldier who had attempted suicide. A kind of strong girdle is first secured around the trunk immediately below the *axillæ;* to this, four small buckles are affixed; two of which are situated anteriorly; two, posteriorly. The head is then encircled by a band which is fastened by means of a buckle at the extremity; and from this band, descend four straps; two of these are crossed behind and secured to the posterior buckles of the girdle; the other two are crossed before; and being drawn through the anterior buckles, depress the chin upon the sternum according to the degree of tightness. The reader, desirous of a more minute knowledge of this bandage, we refer to the description and plate, given of it, in the *Journal Géneral de Medécine, &c. par Sedillot. Tome liv. Page* 105.

Every one conversant with the treatment of transverse wounds of the throat, must have had painful experience of the difficulty with which their margins are kept in contact. Sutures, however strong and numerous, are wholly inadequate to this purpose; they invariably tear out, and only aggravate the cough and fever by the local irritation to which they give rise. To preserve the patient's chin permanently in contact with the sternum is the only way in which the object of the surgeon can be accomplished. The application of common bandages, with this view, will be found utterly ineffectual. Some years since, our attention was strongly attracted to this subject; and we then projected a bandage very nearly resembling that of M. Cavalier; except that, instead of a circular band for the head, we proposed to employ a *cap* made of leather or other strong materials; which would possess the additional security of fastening beneath the chin. The practical advantages of our apparatus, we never since have had an opportunity of determining. EDITORS.

*French Substitute for Cinchona.* The following is the formula of the composition, proposed by Leroy, and employed in France as a substitute for the bark of Cinchona, when, by the ferocious spirit of modern warfare, that valuable exotic had been rendered so scarce as to be accessible only to the opulent. *The French Cinchona,* as it is called, administered in doses one third larger than the *genuine,* is said to have been productive of good effects in bilious, and autumnal intermittent fevers; but not in those of the typhoid type.

Take of madder, Farina of tan (farine de tan), each seven ounces; catechu, one ounce; nut galls two ounces: mix. *Journal de Pharmacie, April* 1816.

---

*Remarks upon an obscure and singular Affection, sometimes occurring in the Female Subject, and very liable to be confounded with Pregnancy; communicated in a Letter to Dr. Palmer.*

C——, June 3d, 1816.

SIR,

ALLOW me, through the medium of the Medico-Chirurgical Journal, to invite the attention of practical men to a subject, of which I have never met with a satisfactory explanation, or scarcely remember ever to have seen noticed by writers. The subject I allude to, is an obscure disease, affecting the female frame, and putting on such a strong character of pregnancy as might delude even the father of physic himself, "the divine old man of Cos," if he had never before met with it. But I dislike circumlocution; and my meaning will be best explained by coming to facts at once.

One spring evening, eight years since, I was summoned abruptly to a woman, whom the messenger reported to be in "strong labour." She lived little more than a mile from our village, and I was soon with her. On my arrival, I found a young woman, of about thirty years, apparently on the brink of confinement of her first child. The gossips were assembled, the baby-linen prepared, and all bespoke anxiety and expectation. After waiting two hours or more, I was disappointed to find that my patient had only little grinding pains at long intervals; and withal so slight, that I could not even propose an examination. With some difficulty, and on the promise of immediately obeying the next summons, I obtained leave from the old women to quit her; and it was afternoon of the next day before I was sent for again. I was now assured, on ascending the stairs, that a little "good help" alone was wanted, and all would be over in a few minutes. The pains, however, were not such as I expected to have found them: they were long in coming on, and cut off very short. At length I took advantage of one stronger than usual, to make an examination. The external parts were, I thought, uncommonly dry and rigid; but what was my amaze, when, upon introducing a finger into the vagina, I found the uterus quite at its superior part, and, to the best of my judgment, *in an unimpregnated state!* I, however, suppressed, as well as I could, every appearance of surprize, and passed my hand very deliberately over every part of the abdomen, which was hard, tense, and very protuberant; but I could discover none of those irregularities which the mature fœtus commonly presents, nor any motion. The breasts were large, and apparently distended with milk. Inquiring farther, I now learnt, that the young woman had been married somewhat more than nine months; that, soon afterwards, her menses had been suppressed, and this change followed by all the usual signs of breeding; that, till within the last three weeks, she had very distinctly felt the motions of the child; but being, at that time, at work in the garden, she had been suddenly taken with a strong shivering fit, and felt a particular coldness of the abdomen, never since entirely leaving her; and that, thenceforward, no motion of the child had been perceptible. This was staggering evidence. I felt myself irresolute and perplexed. Meanwhile, pain after pain succeeded each other, without gaining any thing in strength or frequency; and repeated examinations served only to confirm the opinion I had silently formed, but felt mightily reluctant to avow. At length, having duly prepared my mind for a little rough treatment, I drew the old woman aside, and frankly told her that her daughter was *not* in labour; and, moreover, that I was sure she had never been pregnant. The reception which this bold avowal met with, none but my obstetric brethren can imagine. The company of women was large; and, methought, even the confusion of ancient *Babel* must have been a soothing murmur, when compared to the tumult which now assailed my ears. Abuse descended on me in torrents. Reasoning or remonstrance could be of no avail, for no one would listen. I, therefore, determined to sit down and wait patiently until the clamour had subsided. The old woman now informed me, with many an expression of grinning indignation, that both she and a younger daughter had frequently *seen* the motions of the child, when they were all sitting together near the fire. I know not where the controversy would have ended, had not one of the company, all along more temperate than the rest, fortunately recollected, that, on the morning of the marriage, a reputed *witch*, who lived on a neighbouring heath, had "crossed the path" of the ill-fated couple, and been observed to "mutter to herself." The good woman, hereupon, very considerately added, that, "after all, the gentleman *might* be right." Under cover of this monstrous but friendly superstition, I made good my retreat from the field of conflict with a sound skin; having previously given a dose of *laudanum* to my patient, and directed the abdomen to be frequently fomented and kept regular by *ol. ricini.* A few days afterwards, I called, in going by the woman's door, and was told that her pains were subsiding, and her abdomen getting less: and, in about five weeks, I had the satisfaction to learn, that, without any return of the menstrual flux, or other unusual discharge, every sign of pregnancy had departed.

A second case, of nearly the same kind, fell under my notice in the beginning of 1810. It occurred in the person of our village, aged about 20. Sudden suppression of the menses was followed by protuberance of the abdomen, enlargement of the *mammæ*, great disorder of the stomach, and in short, all the symptoms of the pregnant state. Her situation was soon the topic of the village scandal, and the parish-officer requested my opinion on her case. The poor girl had previously spoken to me about herself; and, sceptical as experience has made me on these subjects, had, by the simplicity and frankness of her manner, nearly dispelled the suspicions which her symptoms might well warrant. But, as the business was likely to take a serious turn, and my character and conduct were very freely handled thereupon, I insisted upon being allowed to make an investigation such as might enable me to decide without fear of mistake. The *os uteri*, and the organ itself, as far as I could examine, were in a condition quite natural; the abdomen large and hard, but without inequality on pressure, or any sense of fluctuation; the breasts turgid. The tongue was much furred; there was loss of appetite and frequent sickness; bowels torpid; urine scanty; pulse feeble and very frequent; spirits much depressed. But the main symptom was constant and distressing pain in the legs and back. My declaration that the girl was not pregnant, was received with much ridicule. She continued to increase in size until her motions were much impeded. Purgatives, light diuretics, and steel, seemed to afford some relief, but nothing more. At length, the swelling became stationary; and, after remaining so some weeks, gradually sunk away without any evident cause whereby it might be explained: and the young woman regained her health and ordinary dimensions to the utter confusion of the infidels, and to the no small amusement and satisfaction of your humble servant.

The third and last case, which happened about the close of the same year, was that of a young and generally healthy unmarried female; in whom all the symptoms of pregnancy supervened on sudden obstruction of the menstrual discharge. She was believed to be about seven months gone when I was requested to see her. After attentive examination *per vaginam*, I declared her *not* pregnant; and I have no doubt of the result: although candour induces me to add, that from the distance which the young woman lived, I had never any after-tidings of her.

I am quite in the dark, Sir, as to the cause of the very singular affection I have tried to give a general idea of; and my object is to request you, or some of your Correspondents, to enlighten me a little upon it. I would just remark, in concluding, that, although the three cases above described happened within so short a space of time, I have not, within more than five years' subsequent practice, met with another instance of it.

I am, Sir, yours, &c.

---

*Note.* I profess myself utterly incompetent to afford my Correspondent any satisfactory explanation of the origin or nature of the phænomenon in question; and beg to solicit the attention of gentlemen to it, whose experience in midwifery-practice may enable them to throw some light upon this obscure affection. I find a case very nearly analogous, in Leroux's *Journal de Medecine* for our next number.

SHIRLEY PALMER.

---

*Cantharides: their chemical analysis; and changes which they exhibit in combination with other substances.* The blistering fly *(meloe vesicatorius Lytta)* consists, according to M. Robiquet, of 1st. a green fluid oil, insoluble in water, soluble in alcohol;—2ndly, a black substance soluble in water but insoluble in alcohol;—3dly a yellow viscous substance, soluble in water and in alcohol at the ordinary temperature (all these three are destitute of the blistering property); 4thly, a white substance, under the form of small crystaline scales, insoluble in water except when combined with the yellow substance; soluble in boiling alcohol, from which it precipitates upon cooling, in crystalline *bracteæ*, after the manner of *spermaceti*, soluble in the oils; and eminently epispastic;—5thly, a fat substance, insoluble in alcohol, not epispastic;—6thly, phosphate of lime, constituting the base of the skeleton;—phosphate of magnesia;—8thly, a small portion of acetic acid; and 9thly, a more considerable quantity of uric acid.

As the cantharides employed by M. Orfila, in his chemical experiments upon this poison, seems to have been prepared with *brandy* instead of alcohol (see page 45 of this number); I thought that it might be useful to repeat the experiments with tincture prepared according to a *British Pharmacopeia*, and state the results concisely to my readers.

The tincture, employed in these experiments, was prepared by myself according to the formula of the *London Pharmacopeia*; and, when filtered, had a beautifully transparent greenish yellow colour.

1. Mixed in the proportion of one part of the *tincture* to four parts of *pure water*, the fluid became of a pale soft amber colour; and remained unaltered, without the slightest precipitation, at the end of twelve hours.

2. No change of colour; no precipitation, on adding it to infusion of litmus.

[*The remaining seven Experiments are deferred till our next Number, for want of room.*]

A new monthly Medical Journal, entitled, *Journal Universel des Sciences Medicales*, edited by Doctor Pariset, has recently appeared in the French Capital. Its pretensions are very high, and its avowed objects most comprehensive. *One* of those objects is a system of *comparative medicine*, the materials for which are *to be* drawn from all the nations on the surface of our globe. The first number has not yet reached us; but if the Editor of the *Bibliothèque Medicale* may be believed, its execution is, by no means, equal to the design; nor does it contain, independently of the Doctor's bombastic introduction, one original article.

Dr. Clough, Physician-Accoucheur to the St. Mary-le-Bonne General Dispensary, and to the Endeavour or Benevolent Society, will commence his Summer Course of Lectures on the Science and Practice of Midwifery, including the Diseases of Women and Children, on Monday morning the 7th of July, at half past ten, and at seven in the evening.

Died.—In the 82d year of his age, Thomas Henry, Esq. the celebrated Chemist, President of the Literary and Philosophical Society of Manchester, F.R.S. &c. &c.—In Princes-street, Leicester-square, James Wilson, Esq. late Surgeon to the Royal African Corps.—

