# Errata

**Published:** 1816-08

**Authors:** 


					87, last line, for cantharides, read tincture of cantharidcs?

				

